# Composition of the alfalfa pathobiome in commercial fields

**DOI:** 10.3389/fmicb.2023.1225781

**Published:** 2023-08-24

**Authors:** Lev G. Nemchinov, Brian M. Irish, Igor V. Uschapovsky, Sam Grinstead, Jonathan Shao, Olga A. Postnikova

**Affiliations:** ^1^Molecular Plant Pathology Laboratory, United States Department of Agriculture, Agricultural Research Service, Beltsville, MD, United States; ^2^Plant Germplasm Introduction and Testing Research Unit, Prosser, WA, United States; ^3^Federal Research Center for Bast Fiber Crops, Tver, Russia; ^4^United States Department of Agriculture, Agricultural Research Service, Office of The Area Director, Beltsville, MD, United States; ^5^Animal Biosciences and Biotechnology Laboratory, Beltsville Agricultural Center, United States Department of Agriculture, Agricultural Research Service, Beltsville, MD, United States

**Keywords:** pathobiome, alfalfa, *Medicago sativa*, high-throughput sequencing, viruses, bacteria, fungi, host response

## Abstract

Through the recent advances of modern high-throughput sequencing technologies, the “one microbe, one disease” dogma is being gradually replaced with the principle of the “pathobiome”. Pathobiome is a comprehensive biotic environment that not only includes a diverse community of all disease-causing organisms within the plant but also defines their mutual interactions and resultant effect on plant health. To date, the concept of pathobiome as a major component in plant health and sustainable production of alfalfa (*Medicago sativa* L.), the most extensively cultivated forage legume in the world, is non-existent. Here, we approached this subject by characterizing the biodiversity of the alfalfa pathobiome using high-throughput sequencing technology. Our metagenomic study revealed a remarkable abundance of different pathogenic communities associated with alfalfa in the natural ecosystem. Profiling the alfalfa pathobiome is a starting point to assess known and identify new and emerging stress challenges in the context of plant disease management. In addition, it allows us to address the complexity of microbial interactions within the plant host and their impact on the development and evolution of pathogenesis.

## 1. Introduction

The productivity of alfalfa, the most extensively cultivated forage legume in the world and the third most widely grown crop in the United States, has often been affected and limited by different diseases, insect pests, and abiotic stress factors (Monteros and Bouton, 2009). Over 70 diseases caused by more than 130 species of different infectious agents, including fungi, Ooymycetes, bacteria, viruses, phytoplasmas, nematodes, and parasitic plants, are described in the latest edition of the Compendium of Alfalfa Diseases and Pests (Samac et al., 2015). In the United States alone, diseases and nematodes of alfalfa cause at least US$400 million and arthropods US$260 million in economic losses annually (Summers, 1998).

Through the recent advances of modern high-throughput sequencing technologies, the “one microbe, one disease” dogma is shifting toward the principle of the “pathobiome”, a diverse community of pathogenic microbes within the biotic environment of the plant host (Vayssier-Taussat et al., 2014; Bass et al., 2019; Mannaa and Seo, 2021). While numerous individual pathogens and diseases they cause in alfalfa have been described, to date, the conception of the alfalfa pathobiome as a major component in plant health and sustainable production is non-existent, and its detailed characterization has not been carried out. Meanwhile, the importance of an organism's pathobiome and understanding of pathogenicity as a final product of complex interactions involving the host, diverse pathogenic communities, and all organismal microbiota is rapidly emerging both in plant and animal health (Bass et al., 2019; Trivedi et al., 2020). One of the primary research aims and a starting point leading in this direction is to describe the structural biodiversity of the relevant pathobiomes and to identify potential threats or novel, unsuspected species (Vayssier-Taussat et al., 2014). This will provide an understanding of how the pathobiome promotes disease, creates an environment for synergisms between microbial pathogens, and contributes to the origination and distribution of novel pathogens.

During the process of identifying novel and emerging viruses infecting alfalfa via high-throughput sequencing (HTS) approaches, we noticed that individual field samples were coinfected with dozens of different pathogens, including bacteria and fungi (Bejerman et al., 2020; Nemchinov et al., 2022). We proposed that this sophisticated multi-pathogenic habitat can affect the behavior of all co-infecting organisms, their accumulation in the host, and, possibly, their transmission rates. In this study, we continued to explore the complexity of alfalfa's microbial environment with an emphasis on the composition of the crop's pathobiome, focusing on bacterial, fungal, and viral infections. We also attempted to estimate the host reaction to a consortium of pathogenic microorganisms by profiling gene expression patterns shaped by the plant pathobiome in the field environment.

## 2. Materials and methods

### 2.1. Plant materials

**Figure 1:**
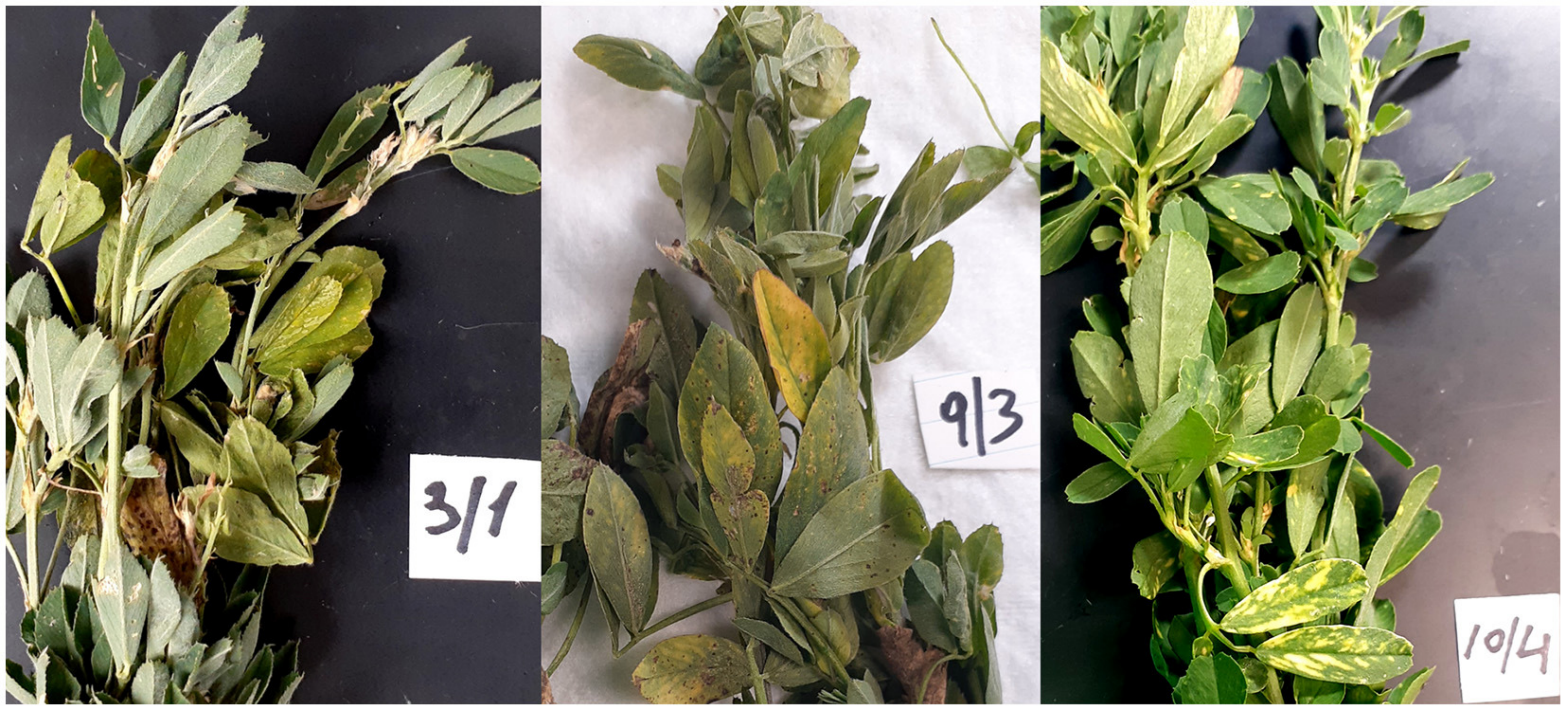
Representative and diverse symptomatology of alfalfa (*Medicago sativa* L.) plant samples collected in the field and used in this study.

Alfalfa samples were collected from 10 commercial fields in Grant County, WA. Specific collection site details were intentionally omitted to protect producers' privacy. Field sampling was done in a zigzag or “W” pattern to include representative samples across the entire field. Two or three plants (the upper 10–15 cm of stems with leaves) were collected from each of the 50 sublocations (five sublocations per field). The symptomatic plants from each sublocation displayed various symptoms shown in Figure 1, including chlorosis, leaf distortions, and leaf spots. These symptoms are often associated with viral and fungal plant pathogens. Since all the commercial fields are in the same geographic area, the symptomatology of the plants across all fields was rather common. The sampling was done on a single day, 6 October 2021, from 8 a.m. to 12 p.m. The age of the fields varied from one to several years of establishment of this perennial crop and would have been late in the harvest season following approximately four cutting/swathing events.

### 2.2. Nucleic acid extraction

Total RNA was extracted from five leaves pooled from the plants in each of the 50 sublocations using the Promega Maxwell^®^ RSC Plant RNA Kit (Promega Corp., Fitchburg, WI, United States) according to the manufacturer's instructions. Total DNA was extracted using the Wizard^®^ Genomic DNA Purification Kit (Promega Corp., Fitchburg, WI, United States).

### 2.3. High-throughput sequencing

Library preparation was performed with Illumina TruSeq Stranded Total RNA with Ribo-Zero kit (Illumina Inc., San Diego, CA, United States), and the sequencing platform used was HiseqX10 (PE150) (Omega Biosciences, Norcross, GA, United States). The 16S and ITS (Internal Transcribed Spacer) library preparation for ribosomal RNA (rRNA) sequencing to 50K, PE300, 25K in each direction, was carried out by Omega Biosciences (Norcross, GA). To discriminate from chloroplast DNA, primers targeted amplification of the DNA encoding V5-V7 region of the bacterial 16S rRNA gene: 799F, 5′ AACMGGATTAGATACCCKG 3′ and 1193R, 5′ ACGTCATCCCCACCTTCC 3′, with Illumina adapters. Primers applied for amplification of the fungal internal transcribed spacer (ITS) region were ITS1F (F) 5′ CTTGGTCATTTAGAGGAAGTAA 3′ and ITS2R (R) 5′ GCTGCGTTCTTCATCGATGC 3′.

### 2.4. Bioinformatics analysis

HTS reads were checked for quality using FastQC 0.11.5 (Andrews, 2010). They were next trimmed of adapters and for quality using Trimmomatic 0.39 (Bolger et al., 2014). The reads were screened against the TruSeq3-PE.fa:2:30:10 adaptors, removing low quality (3) or N bases at the leading and trailing-end of the sequence, scanned with a 4-base sliding window cutting off the quality per base at 15, and reads below 36 were discarded. Trimmed data were assembled by SPAdes (Meleshko et al., 2019) and by QIAGEN CLC Genomics version 21 (QIAGEN, Redwood City, CA, United States). The resulting contigs were screened using BLASTx search against a custom database containing refseq viral protein sequences (https://www.ncbi.nlm.nih.gov/refseq/) and proteomes of *Arabidopsis thaliana* (Uniprot UP000006548) and *Arabidopsis lyrata* (Uniprot UP000008694). The cutoffs for BLASTx were a max target of 50 hits with an expect value of 1. Values > 1e-10 were filtered out. BLASTx was performed on the resulting viral hits against the NCBI nr database for verification. Sequences were further manually screened and filtered for false positives to ensure accuracy. Sequencing coverage depth was calculated by mapping the trimmed reads back to the viral contigs using BBMap 39.01 (Bushnell, 2014). The 16S and ITS sequencing data analyses for bacteria and fungi identification were performed by Omega Biosciences (Norcross, GA) using Illumina BaseSpace 16S Metagenomics Labs app (Version 1.0.0) and RefSeq RDP 16S v3 May 2018 DADA2 and UNITE Fungal ITS Database v7.2.

For metagenomic analysis, adapters were removed, and reads were filtered using bbduck (BBMap; Bushnell, 2014). GNU Parallel 20220222 was used to process the data when possible (Tange, 2018; https://doi.org/10.5281/zenodo.6213471). Trimmed reads were mapped to alfalfa genome (ZhongmuNo.1; Chen et al., 2020) and mitochondria genome of *Medicago truncatula* (KT971339.1). Unmapped reads were merged and submitted to MEGAHIT (Li et al., 2015) for assembly or assembled with SPAdes (Meleshko et al., 2019). Sequences were also screened using MEGAN Huson et al. (2016). For gene expression analysis, the gene subsets of Gene Ontology (GO) terms “response to stress” (GO:0006950) and “photosynthesis” (GO:0015979) were identified from the source data (Chen et al., 2020). To measure the transcript expression levels in the field samples and to eliminate biases related to gene length and sequencing depth, RPKM (reads per kilobase of transcript per million reads mapped) normalization method was used for the calculation of photosynthesis and stress-related genes (Dillies et al., 2013).

### 2.5. Pathogen identification

In addition to the bioinformatics-based analysis of the HTS data, pathogenic species we manually curated using a known list of alfalfa disease-causing organisms (Samac et al., 2015) and based on numerous literature reports from other plant species.

## 3. Results

### 3.1. Snapshot of the microbial community

**Figure 2:**
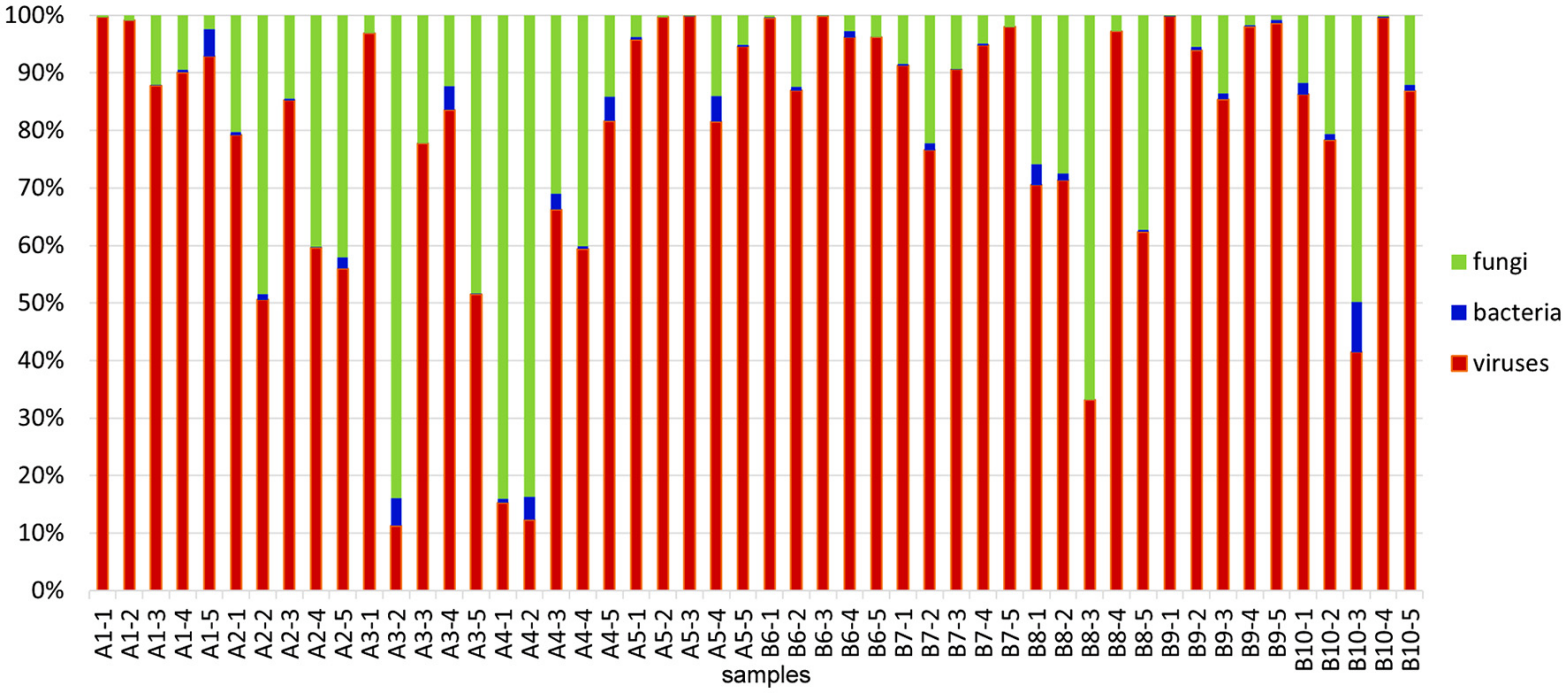
Relative abundances of microbial communities identified in 50 alfalfa plant samples based on the results of RNA-sequencing. Read numbers were obtained by mapping reads back to the assembled contigs from this study that were annotated by Blast. The total microbial count (viruses, bacteria, and fungi) in each sample represents 100%.

The results of RNA sequencing based on the transcripts' expression showed that the dominant constituents of the alfalfa pathobiome are viruses and fungi, while the occurrence of bacterial pathogens was substantially lower (Figure 2). These findings concur with the reports on relatively few bacterial plant pathogens affecting alfalfa, although their impact on the crop is substantial (Getachew et al., 2015).

### 3.2. Alfalfa virome

**Figure 3:**
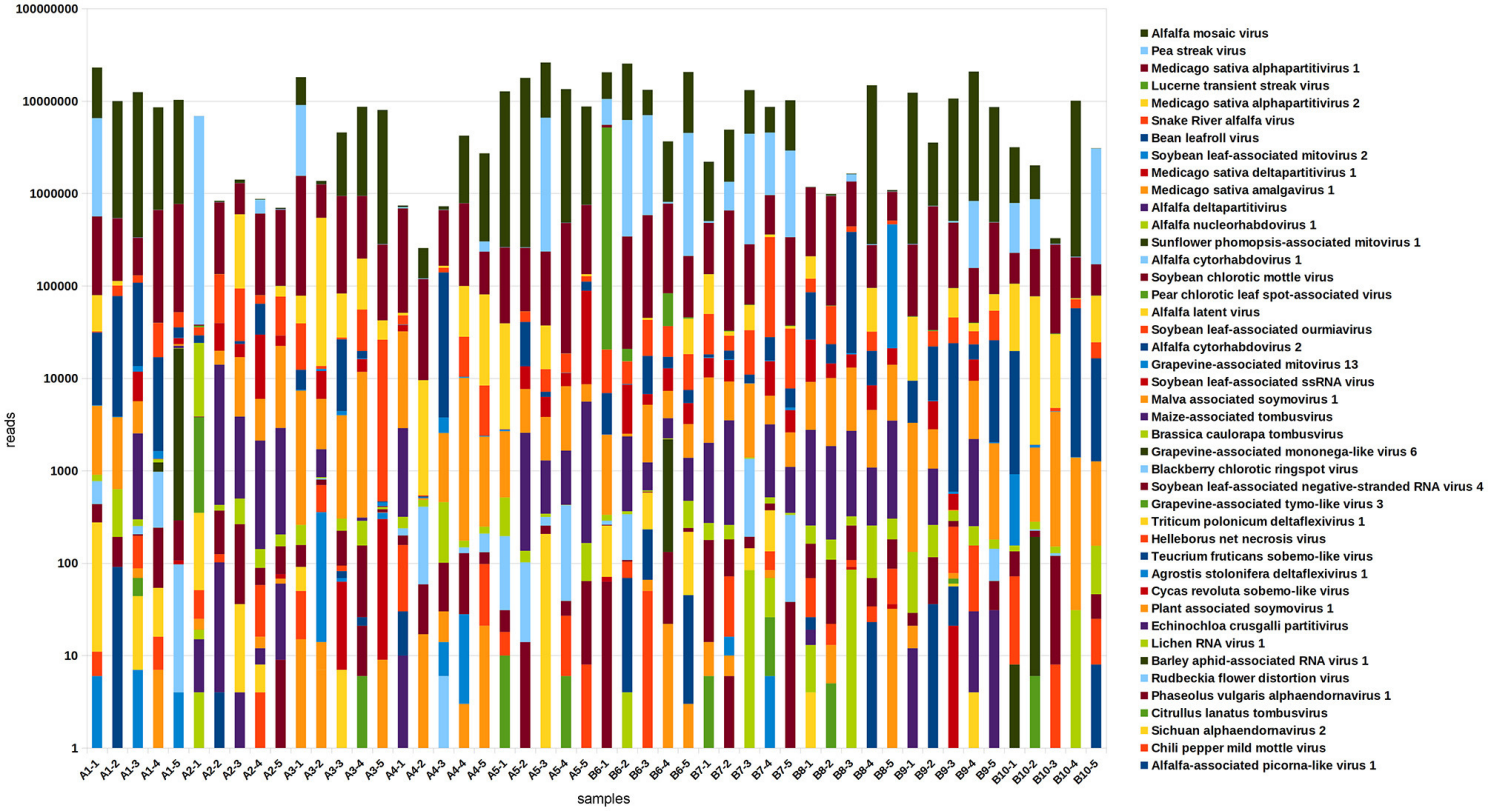
The composition and relative abundance of the alfalfa (*Medicago sativa* L.) virome in 50 plant samples based on the results of RNA-sequencing. Read numbers were obtained by mapping reads back to the assembled contigs from this study that were annotated by Blast.

Viral communities were identified based on results of RNA sequencing. Confirmative with our previous results (Nemchinov et al., 2022), we found that each of the alfalfa samples from commercial fields was coinfected with many different viruses, averaging 10 per sample (Figure 3, Supplementary File 1). Nearly ubiquitous were pathogenic alfalfa mosaic virus, and amalga/partitiviruses that are not commonly recognized to cause disease symptoms in their hosts, although their roles are poorly understood. Several other known viral plant pathogens of alfalfa were found, including lucerne transient streak virus (LTSV), pea streak virus (PeSV), alfalfa latent virus, a strain of PeSV (Nemchinov et al., 2015), and bean leafroll virus (BLRV). Recently discovered, persistent and vertically transmitted Snake River alfalfa virus (SRAV) (Dahan et al., 2022; Postnikova et al., 2023) was detected in all 50 samples. Reads of previously reported three rhabdoviruses (Nemchinov et al., 2022), alfalfa cytorhabdoviruses 1 and 2 (ACRV1&2), and alfalfa nucleorhabdovirus 1 (ANRV1), were identified in majority of the tested samples, indicating that they are widespread in alfalfa, or at least in the surveyed area. In addition, sequencing reads of the following potentially novel viruses or viral strains were revealed:

Blackberry chlorotic ringspot virus (BRSV, estimated 100% identity). The known host range of BRSV, an ilarvirus originally isolated from blackberry (*Rubus* spp.) plants showing diffuse chlorotic spotting and ringspots, does not include *M. sativa* (Jones et al., 2006; Poudel et al., 2014). Notably, there are many feral blackberry plants near the area where alfalfa samples were collected.

Pear chlorotic leaf spot-associated virus (PCLSaV, estimated 34% identity), a novel emaravirus associated with a severe disease of pear (*Pyrus pyrifolia*) trees in China and not identified in alfalfa prior to this work (Liu et al., 2020).

Chili pepper mild mottle virus (CPMMoV, estimated 100% identity), a tobamovirus recently discovered in pepper (*Capsicum* spp.) plants displaying mild mottle and mosaic symptoms (Vélez-Olmedo et al., 2021).

Rudbeckia flower distortion virus (RuFDV, *Caulimoviridae*, estimated 60% identity), a causal agent of severe flower deformation in *Rudbeckia hirta* (Lockhart et al., 2017).

Apple virus E, (AVE, estimated 65–98% identity), an unclassified tombus-like virus isolated from apple (*Malus domestica*), (GenBank: MT892660.1).

Unclassified deltaflexivirus (98–100% identity), (GenBank MN627486), and Phaseolus vulgaris endornavirus 1 (PvEV1, estimated 97% identity), originally isolated from the common bean (*Phaseolus vulgaris)* (Okada et al., 2013) and not reported in alfalfa.

Teucrium fruticans sobemo-like virus (92% identity), originally identified in Jiangsu Province, China (Yang B. et al., 2022; Yang S. et al., 2022).

Some of these viruses, previously unreported in alfalfa, occurred sporadically, in one or two samples (BRSV, PCLSaV, CPMMoV, and RuFDV), while others were identified more frequently (AVE and deltaflexivirus). In total, 24 viruses were identified in all tested samples.

### 3.3. Bacterial communities

**Figure 4:**
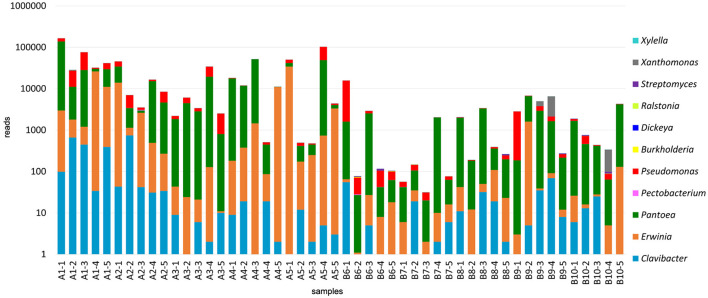
Bacterial communities identified in the 50 alfalfa (*Medicago sativa* L.) plant samples based on the results of 16S V5-V7 amplicon sequencing.

Bacterial communities were identified based on the results from the 16S amplicons. Plant pathogenic bacteria belonging to the following genera were found in the analyzed alfalfa samples: *Burkholderia, Erwinia, Clavibacter, Dickeya, Pantoea, Pectobacterium, Pseudomonas, Ralstonia, Streptomyces, Xanthomonas*, and *Xylella* (Figure 4, Supplementary File 2). Bacterial species of all these genera, except *Ralstonia*, have been well-documented in alfalfa. It is known, however, that the model legume plant *Medicago truncatula*, a close relative of alfalfa, is susceptible to *R. solanacearum* (Vailleau et al., 2007). Importantly, the very few sequencing reads of *Ralstonia* spp. found in this study, may not necessarily represent an authentic infection (Supplementary Table 2). The same is true for *Dickeya* and *Xylella*.

Some bacterial pathogens were found at the species level, including *Clavibacter michiganensis, Erwinia billingiae, Erwinia oleae, Erwinia tasmaniensis, Pantoea agglomerans, Pseudomonas flavescens, Pseudomonas graminis*, and *Pseudomonas viridiflava*. *C. michiganensis* is a cause of bacterial wilt in alfalfa, occurring throughout alfalfa-growing regions (Ophel-Keller, 2015). It was found in 31 of 50 tested samples. *Pseudomonas viridiflava* was reported to cause bacterial stem blight (Lipps et al., 2019) and root rot disease in alfalfa (Heydari et al., 2012). Sequencing reads mapped to this species were found in six samples. The ubiquitous gram-negative *Pantoea agglomerans*, also known as *Erwinia herbicola*, is abundant in plant and animal products and can cause sprout rot in alfalfa (Moline and Kulik, 1997). It was present in all tested samples. The remaining species are not known to cause diseases in alfalfa, although they may be pathogenic in other plant hosts (Hildebrand et al., 1994; Vidaurre-Barahona et al., 2021) or recognized for their biocontrol activities.

### 3.4. Fungal communities

**Figure 5:**
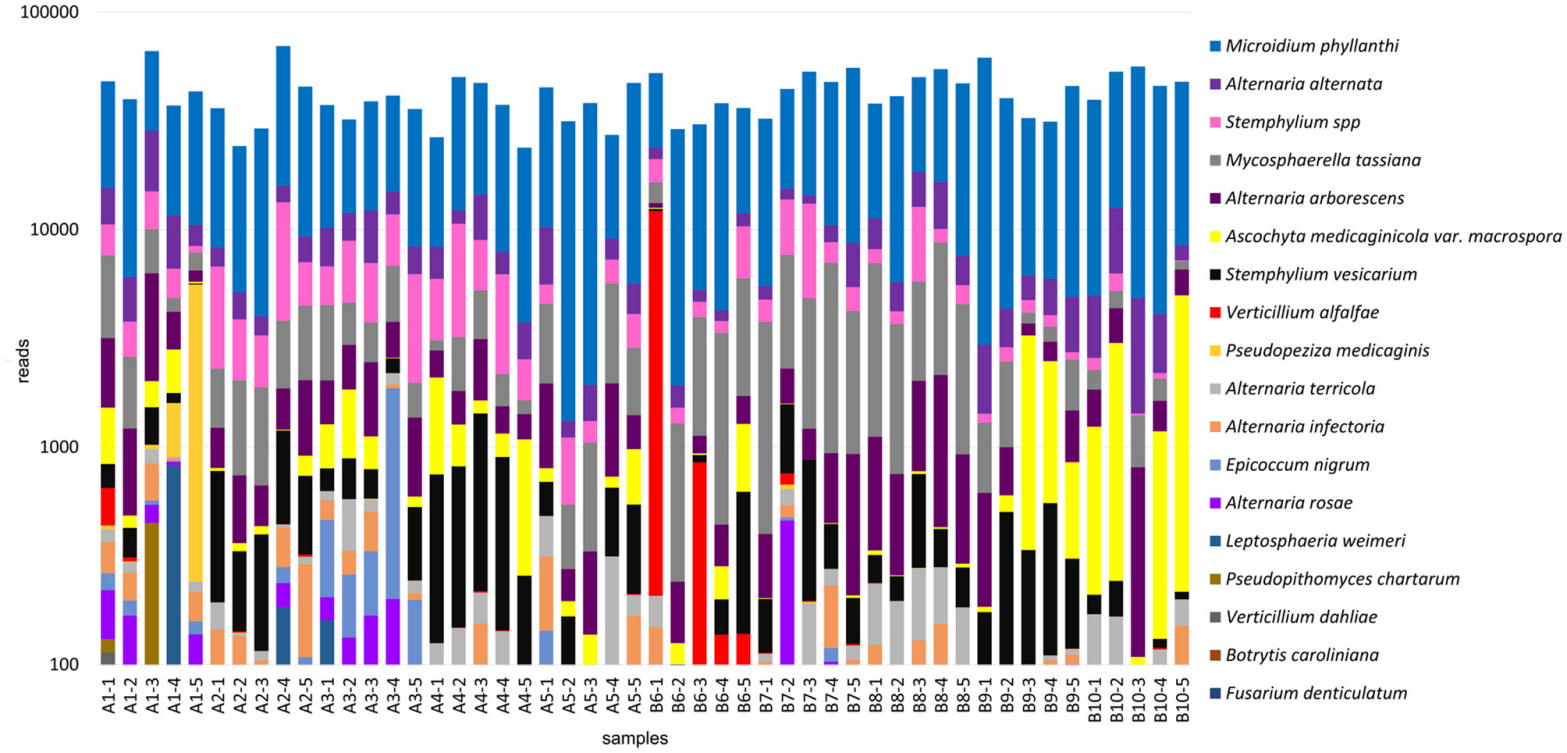
Fungal communities identified in the 50 alfalfa (*Medicago sativa* L.) plant samples based on the results of ITS1-2 amplicon sequencing.

Fungal communities were identified based on the results from the ITS amplicons. Fungal species from 18 genera were identified, including agriculturally important plant pathogens and those not previously reported in alfalfa (Figure 5, Supplementary Table 3). The first group contained several *Alternaria* spp., among which was *A. alternata*, recently found to cause blight symptoms in alfalfa in Canada and also identified in the U.S. alfalfa (Abbasi et al., 2018; Nemchinov et al., 2022); *Pseudopeziza medicaginis*, a causal agent of common leaf spot in alfalfa (Samac et al., 2015); *Verticillium alfalfa* and closely related fungus *V. dahliae*, causing Verticillium wilt in alfalfa (Stuteville and Erwin, 1990; Huang, 2015); *Stemphylium vesicarium*, a causal agent of Stemphylium leaf spot in alfalfa (Smith, 2015); *Ascochyta medicaginicola*, infecting alfalfa and its close relative *M. truncatula* (Chen et al., 2015); *Leptosphaeria weimeri*, the teleomorph of *Stagnospora melioti* causing *Stagnospora* leaf spot and root rot disease (Irwin and Armour, 2015); *Alternaria arborescens* that was recently identified by HTS in the U.S. alfalfa samples from the same growing region (Postnikova et al., 2023).

The second group contained *Mycosphaerella tassiana*, found in *Canabis sativa* and rye (*Secale cereale* L.) and pathogenic in >35 species other than alfalfa (https://species.nbnatlas.org/species/NBNSYS0000016449); *Epicoccum nigrum*, a widespread fungus, an opportunistic pathogen, and a known biocontrol agent (Bruton et al., 1993; Ogórek et al., 2020); *Fusarium denticulatum*, which is known to occur only on sweet potatoes (Nirenberg and O'Donnell, 1998) and was not, to the best of our knowledge, reported in alfalfa; *Botrytis caroliniana* that was recently isolated from blackberry fruits symptomatic for gray mold (Li et al., 2012); *A. rosae, A. infectoria*, and *A. terricola* environmental fungi that could also be pathogenic in certain hosts (Woudenberg et al., 2013; Moslemi et al., 2017) but were not, to the best of our knowledge, reported in alfalfa prior to this study; *Pseudopithomyces chartarum*, pathogenic in wheat (Tóth et al., 2007); *Microidium phyllanthi*, a cause of Phyllanthus powdery mildew on *Phyllanthus reticulatus* black-honey shrub and other Phyllanthus species (Pan et al., 2020), that was not, to the best of our knowledge, described in alfalfa.

Notably, similarly to viruses and bacteria identified by HTS, sequencing reads mapped to some fungal species were relatively abundant in all analyzed samples (*M. phyllanthi, A. alternata, Stemphylium* spp., and *M. tassiana*), while others were sparse (*F. denticulatum, B. caroliniana*, and *L. weimeri*) and thus may not represent authentic infections.

### 3.5. Pathobiome of the selected samples

**Figure 6:**
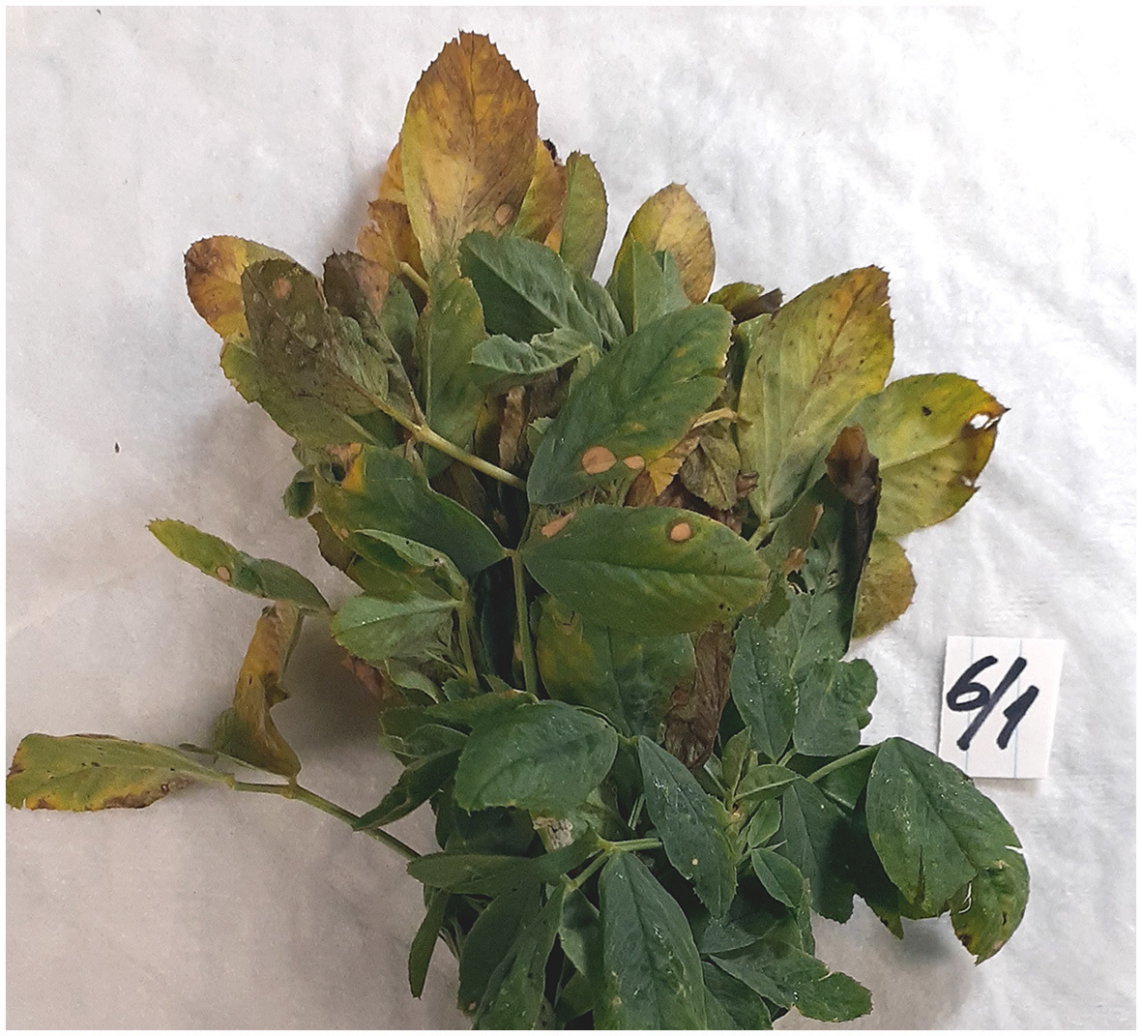
Severe symptoms on what likely was a multiple pathogen infection on an individual alfalfa (*Medicago sativa* L.) plant sample.

On average, each of the alfalfa samples contained 20–30 different pathogens, emphasizing the importance of the complex microbial interactions in affecting plant health. To demonstrate the diversity of microorganisms constituting the pathobiome in individual alfalfa plants, we have chosen three representative samples based on their large and heterogeneous pathogenic communities (Table 1). Some of these alfalfa plant samples displayed diverse, occasionally acute symptomatology (Figure 6). The number of different pathogens in these samples were 37 (A1-1), 25 (A2-1), and 28 (B1-6). According to the RNA-seq estimate, the prevalent pathogens in the samples A1-1 and B6-1 were viruses, mostly AMV and PeSV, while in the sample A2-1, those were viruses (mostly PeSV) and fungi (primarily *Microidium phyllanthi*) (Figure 2, Supplementary Files 1–3).

**Table 1:** Heterogeneity of pathogenic communities in three representative alfalfa plants (*Medicago sativa* L.) plants.

**Samples/Spp**.		
**A1-1**	**A2-1**	**B6-1**
*Microidium phyllanthi*	*Microidium phyllanthi*	*Microidium phyllanthi*
*Alternaria alternata*	*Stemphylium* spp.	*Verticillium alfalfae*
*Mycosphaerella tassiana*	*Alternaria alternata*	*Stemphylium* spp.
*Stemphylium* spp.	*Mycosphaerella tassiana*	*Mycosphaerella tassiana*
*Alternaria arborescens*	*Stemphylium vesicarium*	*Alternaria alternata*
*Ascochyta medicaginicola var. macrospora*	*Alternaria arborescens*	*Alternaria arborescens*
*Verticillium alfalfae*	*Alternaria infectoria*	*Stemphylium vesicarium*
*Stemphylium vesicarium*	*Alternaria rosae*	*Ascochyta medicaginicola var. macrospora*
*Alternaria infectoria*	*Alternaria terricola*	*Alternaria infectoria*
*Alternaria rosae*	*Epicoccum nigrum*	*Alternaria terricola*
*Verticillium dahliae*	*Ascochyta medicaginicola var. macrospora*	*Alternaria rosae*
*Alternaria terricola*	*Pseudopithomyces chartarum*	*Epicoccum nigrum*
*Epicoccum nigrum*	*Pantoea* spp.	*Botrytis caroliniana*
*Fusarium denticulatum*	*Erwinia* spp.	*Pseudopithomyces chartarum*
*Botrytis caroliniana*	*Pseudomonas* spp.	Alfalfa mosaic virus
*Pseudopithomyces chartarum*	*Clavibacter* spp.	Bean leafroll virus
*Pseudopeziza medicaginis*	*Xanthomonas* spp.	Pea streak virus
Alfalfa latent virus	*Pectobacterium* spp.	*Echinochloacrus gallipartitivirus*
Alfalfa mosaic virus	*Xylella spp*.	*Medicago sativa alphapartitivirus 1*
Bean leafroll virus	*Dickeya* spp.	*Medicago sativa alphapartitivirus 2*
Pea streak virus	Pea streak virus	*Snake River alfalfa virus*
*Echinochloacrus gallipartitivirus*	Alfalfa latent virus	*Clavibacter* spp.
Medicago sativa alphapartitivirus 1	Alfalfa mosaic virus	*Erwinia* spp.
Medicago sativa alphapartitivirus 2	Snake River alfalfa virus	*Pantoea* spp.
Medicago sativa amalgavirus 1	Bean leafroll virus	*Pseudomonas* spp.
Soybean leaf-associated mitovirus 2		*Burkholderia* spp.
Alfalfa deltapartitivirus		*Streptomyces* spp.
Snake River alfalfa virus		*Xylella* spp.
*Pantoea* spp.		
*Pseudomonas* spp.		
*Erwinia* spp.		
*Xanthomonas* spp.		
*Clavibacter* spp.		
*Streptomyces* spp.		
*Pectobacterium* spp.		
*Burkholderia* spp.		
*Dickeya* spp.		

### 3.6. Field pathobiome and host genomics

**Figure 7:**
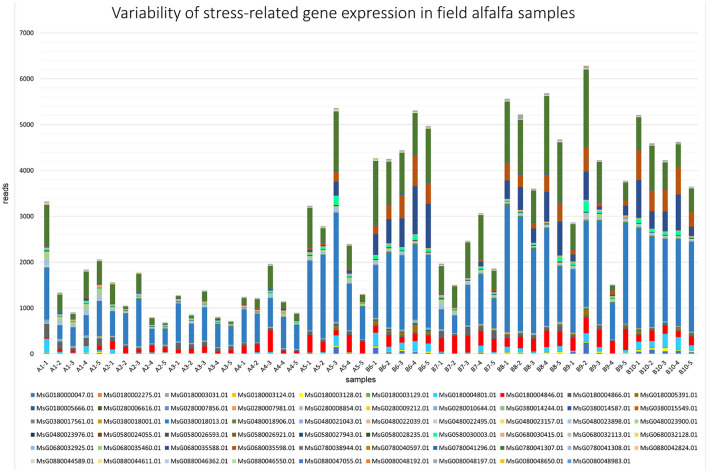
Variability of expression patterns in stress-related genes in field alfalfa (*Medicago sativa* L.) samples.

**Figure 8:**
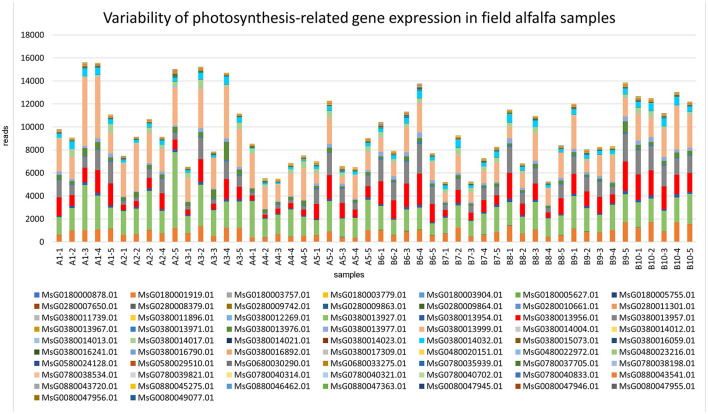
Variability of expression patterns in photosynthesis-related genes in field alfalfa (*Medicago sativa* L.) samples.

Comparatively to the field pathogenomics strategy that was introduced to provide insight into the population structure of emerging plant pathogens in the field (Hubbard et al., 2015), we used a similar approach except for a survey of host GE patterns shaped by the plant pathobiome in the field environment. Since all plants in commercial alfalfa fields are to some degree impacted by various organisms, it is not possible to obtain *de facto* uninfected control samples for differential GE analysis. For that reason, we assessed the expression of the selected groups of genes quantitatively by mapping sequencing reads to the genome of *M. sativa* (Chen et al., 2020). The selected categories were stress-and photosynthesis-related genes, annotated by their respective Gene Ontology (GO) IDs (Chen et al., 2020). The expression of both groups of genes was notably fluctuating between different samples, although no definite correlation was established with the presence of specific pathogens (Figures 7, 8).

Among stress-related genes, dehydrins (MsG0380018013.01, MsG0780041307.01, MsG0680035598.01, and MsG0680035588.01) were the most prominently expressed in all samples (Figure 7), particularly in fields 8 and 10, reaching over 50,000 reads in sample 8-2. While the response of plant dehydrins of the LEA (late embryogenesis abundant) protein family to abiotic stress is largely documented (Liu et al., 2017), their pleotropic effect, including on biotic stress tolerance, has also been reported (Brini et al., 2011). Besides dehydrins, expression levels of genes encoding heat shock proteins (MsG0580030003.01), critical for plant defense responses (Lee et al., 2012), varied substantially. The variability in expression of other genes regulating resistance mechanisms to biotic and abiotic stresses was notable as well (calmodulin, MsG0480023900.01, and universal stress protein, MsG0680030415.01).

Among the photosynthesis-related genes (Figure 8), transcripts mapped to the genes encoding photosystem II (PSII) D2 protein (MsG0380014032.01), which forms the reaction core of PSII; reaction center protein H (MsG0380013999.0), required for stability and assembly of the core complex of PSII; PSII reaction center PsbK protein (MsG0380013927.01), a component of PSII; and photosystem I (PSI) PsaA/PsaB proteins (MsG0380013957.01), central to the electron transfer from plastocyanin to ferredoxin (Paysan-Lafosse et al., 2023), were among the most abundant and variable. The sensitivity of photosynthetic processes to pathogen infection is well-known (Yang and Luo, 2021).

## 4. Discussion

In this study, we have attempted to characterize by HTS technologies a community of pathogenic organisms, or a pathobiome, of alfalfa, a widely cultivated legume forage crop in many countries around the world. The primary focus of this initial research was on viral, bacterial, and fungal infections. We have also evaluated the variability of gene expression in alfalfa field samples to predict the potential impact of the pathobiome on host fitness and its role as a selective agent in plant evolution. There are several important conclusions that can be drawn from the results of this study:

In field conditions, alfalfa plants are coinfected with a diverse mix of known disease-causing organisms as well as novel, potentially pathogenic agents. It is therefore objectionable to describe field alfalfa diseases strictly based on the “one pathogen, one disease” dogma. Even though a single pathogen could be implicated in the development of a characteristic disease symptomatology in laboratory conditions, a highly diverse, interacting community of microorganisms inhabiting alfalfa plants in the field unquestionably contributes to the health status of the crop.

Plant viruses signify a ubiquitous community of the alfalfa pathobiome, essentially contributing to its diversity and likely to all aspects of complex interactions between coinfecting pathogens and the host. Many of these viruses are known to cause diseases in alfalfa, while the pathogenicity of others has not been established yet and requires further investigation. Importantly, alfalfa may potentially acquire viruses from non-legume hosts and serve as a reservoir for their dissemination, as could happen in the case of BRSV and other viruses found in this study.

The occurrence of the bacterial pathogens in the examined alfalfa samples was not extensive, although sequencing reads from some of the major pathogenic genera were identified, including *C. michiganensis*, a cause of bacterial wilt throughout the alfalfa-growing regions and a serious problem for the U.S. alfalfa (Ophel-Keller, 2015). It is known that relatively few bacterial pathogens, as compared to viral and fungal infections, affect alfalfa in field conditions (Getachew et al., 2015).

Fungal infections, along with viruses, were dominant components of the alfalfa pathobiome in the examined alfalfa plants and included known agriculturally important pathogens as well as those not previously reported in alfalfa. Similar to the bacterial and viral agents with undefined pathogenic status in alfalfa, the latter group must be investigated experimentally to establish its pathogenic potential.

An evident variability of gene expression was found in alfalfa plants infected with different groups of microorganisms in the field environment. To the best of our knowledge, this “field host genomics” approach to survey host GE responses to cumulative pathogens in the field through high-throughput transcriptomics is a novel undertaking that can offer genomic insights into resistance to multi-pathogenic infections for acceleration of breeding programs. It is therefore likely that a field pathobiome can be a powerful selective agent in plant evolution, affecting host fitness and agricultural traits (Kover and Schaal, 2002). However, distinct criteria for shaping host genomic responses to the collective pathobiome in the field must be developed, including comparability of the data with uninfected controls and an accurate relationship between different components of the pathobiome and patterns of host gene expression.

## Data availability statement

The datasets presented in this study can be found in online repositories. The names of the repository/repositories and accession number(s) can be found below: https://www.ncbi.nlm.nih.gov/bioproject; PRJNA975554.

## Author contributions

LN: concept, data analysis, and first draft of the manuscript. BI: survey, sample collection, and evaluation. IU: data analysis. SG and JS: bioinformatics and data analysis. OP: selection of the HTS methodologies, bioinformatics, and data analysis. All authors contributed to the editing of the manuscript and approved it for publication.

## Supplementary material

Supplementary File 1 The number of sequencing reads in examined alfalfa (*Medicago sativa* L.) samples that mapped to different plant viruses.

Supplementary File 2 The number of sequencing reads in examined alfalfa (*Medicago sativa* L.) samples that mapped to different pathogenic bacteria.

Supplementary File 3 The number of sequencing reads in examined alfalfa (*Medicago sativa* L.) samples that mapped to different pathogenic fungi.

Supplementary File 4 Variability of expression patterns in stress- and photosynthesis-related genes in field alfalfa (*Medicago sativa* L.) samples.
